# Systematic Review of Artificial Intelligence for Abnormality Detection in High-volume Neuroimaging and Subgroup Meta-analysis for Intracranial Hemorrhage Detection

**DOI:** 10.1007/s00062-023-01291-1

**Published:** 2023-06-01

**Authors:** Siddharth Agarwal, David Wood, Mariusz Grzeda, Chandhini Suresh, Munaib Din, James Cole, Marc Modat, Thomas C Booth

**Affiliations:** 1 School of Biomedical Engineering & Imaging Sciences, King’s College London, Rayne Institute, 4th Floor, Lambeth Wing, SE1 7EH London, UK; 2 Leicester Medical School, University of Leicester, LE1 7RH Leicester, UK; 3 Centre for Medical Image Computing, Department of Computer Science, University College London, WC1V 6LJ London, UK; 4 Department of Neuroradiology, Ruskin Wing, King’s College Hospital NHS Foundation Trust, SE5 9RS London, UK

**Keywords:** Machine learning, Deep learning, Anomaly detection, Clinical validation, Brain MRI

## Abstract

**Purpose:**

Most studies evaluating artificial intelligence (AI) models that detect abnormalities in neuroimaging are either tested on unrepresentative patient cohorts or are insufficiently well-validated, leading to poor generalisability to real-world tasks. The aim was to determine the diagnostic test accuracy and summarise the evidence supporting the use of AI models performing first-line, high-volume neuroimaging tasks.

**Methods:**

Medline, Embase, Cochrane library and Web of Science were searched until September 2021 for studies that temporally or externally validated AI capable of detecting abnormalities in first-line computed tomography (CT) or magnetic resonance (MR) neuroimaging. A bivariate random effects model was used for meta-analysis where appropriate. This study was registered on PROSPERO as CRD42021269563.

**Results:**

Out of 42,870 records screened, and 5734 potentially eligible full texts, only 16 studies were eligible for inclusion. Included studies were not compromised by unrepresentative datasets or inadequate validation methodology. Direct comparison with radiologists was available in 4/16 studies and 15/16 had a high risk of bias. Meta-analysis was only suitable for intracranial hemorrhage detection in CT imaging (10/16 studies), where AI systems had a pooled sensitivity and specificity 0.90 (95% confidence interval [CI] 0.85–0.94) and 0.90 (95% CI 0.83–0.95), respectively. Other AI studies using CT and MRI detected target conditions other than hemorrhage (2/16), or multiple target conditions (4/16). Only 3/16 studies implemented AI in clinical pathways, either for pre-read triage or as post-read discrepancy identifiers.

**Conclusion:**

The paucity of eligible studies reflects that most abnormality detection AI studies were not adequately validated in representative clinical cohorts. The few studies describing how abnormality detection AI could impact patients and clinicians did not explore the full ramifications of clinical implementation.

**Supplementary Information:**

The online version of this article (10.1007/s00062-023-01291-1) contains supplementary material, which is available to authorized users.

## Introduction

In the developed world, first-line imaging is performed in almost all hospitals, and refers to imaging performed at onset, for example, a head computed tomography (CT) for an unconscious patient in the emergency department, or a head magnetic resonance imaging (MRI) for a patient with headache. First-line imaging is a high-volume task and a range of pathologies can be encountered. We distinguish this from second-line imaging where detailed biomarkers are extracted, based on prior clinical and first-line imaging information. Typically, second-line imaging is only performed in specialist hospitals where examples include large vessel occlusion imaging for stratifying stroke patients for thrombectomy treatment, or perfusion imaging for characterising brain tumours [1]. In comparison to first-line imaging, second-line imaging is a low-volume task.

Radiology workloads for first-line imaging have soared in the last decade due to changing demographics, increased screening for early diagnosis initiatives, and updated clinical pathway guidelines requiring imaging. In the years leading up to the coronavirus disease 2019 (COVID-19) pandemic, the number of brain MRI scans performed in, for example, the United Kingdom (UK) increased on average by 7.8% annually, and the demand for both CT and MRI reporting outpaced the growth in the radiology workforce [2, 3]. Reporting backlogs are problems of national importance in the UK, and analogous scenarios are seen in healthcare systems globally. Diagnostic delays cause poorer short and long-term clinical outcomes, with the late detection of illness inflating healthcare costs [4].

The automated detection of abnormalities in a scan using artificial intelligence (AI) has the potential to improve radiologist efficiency. The AI can be used to reorder radiology worklists by flagging abnormal scans, as a reader aid or even as a second reader to identify missed pathology. However, the considerable interest in introducing AI into clinical environments to improve productivity in the high volume first-line imaging tasks, may be clouded by two main challenges in most published studies. Firstly, many abnormality detection AI studies report the diagnostic accuracy using non-representative clinical datasets (e.g. intracranial hemorrhage alone versus healthy controls without any other pathology) [5], including commercially available AI solutions [6]. Indeed, few studies validate their findings on datasets that are representative of the scans seen in routine clinical practice which contain a wide variety of pathologies. A second concern is that many studies do not demonstrate the generalisability of AI models due to inadequate validation methodology [7, 8]. By validating abnormality detection AI on a hold-out subset from the same patient dataset, known as internal validation, it is unclear whether reported AI performance would translate to different patient populations scanned at different institutions. A recent systematic review analysing the enormous number of recent studies where AI was used for the detection of COVID-19 using chest imaging, found that all 62 included studies had no potential clinical use due to methodological biases such as the use of unrepresentative datasets and insufficient validation [9].

The aim of this systematic review was to determine the diagnostic performance and summarise the evidence supporting the use of those AI models carrying out first-line neuroimaging tasks. Critically, we ensured that our analyses were only focused on those studies that were not compromised by unrepresentative datasets or inadequate validation methodology. Therefore, we analysed those AI models that might conceivably be ready for use in the clinic. The primary objective was to determine the diagnostic accuracy of these AI models. A secondary objective was to determine the impact of AI on downstream clinical outcomes in those studies where this had been investigated.

## Methods

This systematic review was conducted in accordance with the Preferred Reporting Items for Systematic Reviews and Meta-Analyses of Diagnostic Test Accuracy (PRISMA-DTA) statement [10]. The review protocol is registered on the international prospective register of systematic reviews (PROSPERO), CRD42021269563.

### Data Sources and Searches

The full strategy is listed in Supplementary Material 1. Searches were conducted on MEDLINE, EMBASE, the Cochrane Library and Web of Science for studies published until September 2021. Bibliographies from eligible studies and systematic reviews were searched for additional relevant studies. Conference abstracts and pre-prints were excluded. A full description of data extraction is provided in Supplementary Material 2.

### Index Test, Reference Standard and Target Condition

The target condition of the systematic review was the abnormality detected, for example intracranial hemorrhage. The AI model detecting the target condition was the index test. The radiological review was designated as the reference standard.

### Inclusion Criteria

We included studies where an AI model could predict if a given CT or MRI examination was abnormal. Only studies that validated AI models on test datasets that were separated from the training data temporally or geographically were included. Test datasets were required to have normal scans, scans with the target condition and scans with one or more non-target conditions, in order to be representative of clinical practice.

### Exclusion Criteria

The motivation for the study was to review abnormality detection in first-line, clinical neuroimaging. Studies that only reported the accuracy of the AI model to make voxel-wise (e.g. segmentation studies) or slice-wise predictions but did not subsequently report at the examination level were excluded (unless examination level accuracy could be calculated from the published study data). Studies using second-line imaging exclusively (e.g. angiography, perfusion studies) were excluded. Psychiatric conditions were excluded if structural differences have only been shown in group-wise comparison studies (e.g. schizophrenia, autism spectrum disorder); all conditions that had a structural correlate often seen at the individual level were included (e.g. Alzheimer’s disease). Studies testing exclusively on pediatric populations were excluded. Studies not published in a peer-reviewed journal or without an English language translation were excluded [11].

### Data Analysis

We used the QUality Assessment of Diagnostic Accuracy Studies‑2 (QUADAS-2) tool [12], tailored to the review question incorporating items from the Checklist for Artificial Intelligence in Medical Imaging (CLAIM) [13]; modified signalling questions are presented in Supplementary Material 3. The unit of analysis was the patient undergoing a CT or MRI examination. The primary outcome was diagnostic test accuracy. Secondary outcomes assessed whether the AI model had been implemented in clinical practice (i.e. in a pathway that could affect clinical outcomes rather than reporting diagnostic test accuracy on a retrospective test dataset in a dry laboratory setting), and if so, the associated performance metrics.

To determine the primary outcome measures, where published, the 2 × 2 contingency tables and the principal diagnostic accuracy measures of sensitivity (recall) and specificity were extracted for test datasets. The area under the receiver operating characteristic curve (ROC-AUC) values, and positive predictive values (PPV or precision) were also extracted where published. Where 2 × 2 contingency tables were not provided, the tables were populated based on the published study data; the calculations are outlined in Supplementary Material 4.

The PPV is important for abnormality detection (Supplementary Material 4) and is more informative than specificity for imbalanced datasets particularly when the prevalence of the target condition is small [14]. The calculation of PPV is, however, dependent on the prevalence of the target condition in a test dataset, where PPV increases with increasing prevalence assuming constant sensitivity and specificity; the calculation is outlined in Supplementary Material 4 [15].

To directly compare AI model performance, the PPV for each model must be adjusted for a uniform prevalence. There were sufficient studies in one subgroup (intracranial hemorrhage detection in CT scans) for the calculation of prevalence-adjusted PPV. Here, we chose a prevalence of 10% based on recent evidence of routine clinical practice in the UK [16]. The prevalence-adjusted PPV we subsequently quote can be interpreted as the PPV that would be expected for each model if the prevalence of ICH within the test dataset was 10%.

### Meta-analysis

Meta-analysis was performed when four or more studies evaluated a given target condition within a specific modality [17]. Studies investigating the detection of intracranial hemorrhage on CT scans were the only subgroup of sufficient number and homogeneity to allow inclusion for meta-analysis.

A bivariate random effects model was used for meta-analysis, taking into account the within and between study variance, and the correlation between sensitivity and specificity across studies [18]. Sensitivities and specificities were presented for each study using forest plots, and pooled estimates for both measures were calculated. To investigate the impact of variables of interest contributing to heterogeneity, metaregression was performed with the variable of interest as a covariate for the bivariate model. Using the existing model parameters, the absolute differences in pooled sensitivity and specificity between subgroups of interest were computed.

Parameters of the model also allowed the estimation of the summary ROC (SROC) curve and the summary ROC-AUC (SROC-AUC). Using a resampling approach [19], the model estimates were also used to derive the pooled measures of balanced accuracy as well as the positive and negative likelihood ratios and the diagnostic odds ratio.

The meta-analysis was conducted by a statistician (M.G.) with 15 years of relevant experience. All the statistical analyses were performed in R (v 3.6.1, R Foundation for Statistical Computing, Vienna, Austria). The R package mada (v 0.5.10) was used for the bivariate model [20]. As some of the 2 × 2 contingency table input cell values derived from the individual studies contained zeros, we applied a continuity correction (0.5).

## Results

### Characteristics of Included Studies

Database searches resulted in 42,870 unique results, of which 5734 potentially eligible full texts were assessed (Supplementary Fig. 1). Our criteria for clinically representative test datasets were not met in 1239 studies which were excluded. Additionally, we excluded 218 studies for using internal validation only. Only sixteen studies were of sufficient scientific rigour to be eligible for inclusion. The test datasets from the 16 studies comprised of 26,164 patients in total; however, the total number of patients in the training datasets could not be calculated as some commercial studies did not publish this data. Supervised convolutional neural networks (CNNs) to classify scans as normal or abnormal were used in 14/16 (88%) studies, with a variety of different model architectures and 5 studies (5/16, 31%) demonstrated the accuracy of commercially available AI models (from three AI vendors: Qure.ai (Mumbai, India); Aidoc (Tel Aviv, Israel); Avicenna.ai (La Ciotat, France) [21–26]). The largest subgroup of studies (11/16, 69%) employed CNNs to detect intracranial hemorrhage using CT [21–25, 27–32]. Other studies used CT and MRI to detect other single non-hemorrhage pathology (2/16, 13%) [21, 33], or multiple pathologies (4/16, 25%) [33–36]. The characteristics of each included study are summarised in Supplementary Material 5 with further AI model information shown in Table 1.

**Fig. 1:**
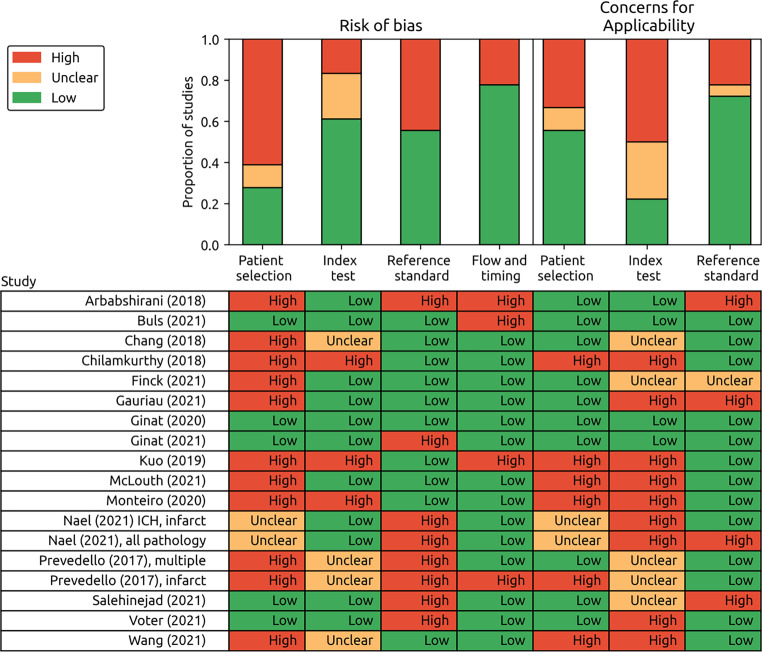
Summary of the QUADAS‑2 risk of bias assessment

**Table 1:** Summary characteristics of AI models for each study. Machine learning models typically use training labels in order to establish a relationship between medical images and the model outputs. Further details regarding the training and test sets can be found in Supplementary Material 5

Study (author, year)	Modality	Target pathology	Index test	Training labels	Model outputs
Arbabshirani (2018) [32]	CT	ICH	CNN, with 5 convolutional and 2 fully connected layers	Examination-level binary presence of abnormality (present/not present)	Binary prediction of ICH (present/not present) for each examination
Buls (2021) [24]	CT	ICH	Aidoc v1.3, a proprietary CNN	Combination of examination-level binary labels, bounding boxes and segmentations	Binary prediction of ICH (present/not present) for each examination, key images for review
Chang (2018) [31]	CT	ICH	CNN, modified mask R‑CNN architecture	Manual segmentations for each examination	Binary prediction of ICH (present/not present) for each examination, segmentations, and volume estimation of ICH
Chilamkurthy (2018) [21]	CT	ICH	Qure.ai proprietary CNN, modified ResNet18 architecture	Slice-level binary presence of abnormality (present/not present)	Binary prediction of ICH (present/not present) for each examination
	CT	Mass effect			
Chilamkurthy (2018) [21]	CT	Skull fracture	Qure.ai proprietary CNN, modified DeepLab architecture	Bounding-box annotations per slice	
Finck (2021) [34]	CT	Any pathology	“Weakly supervised machine learning”: normative learning by registering normal brains to a shared atlas and determining per-voxel confidence-intervals	Not directly trained on labels, although training was conducted on brains that were known to be normal	Prediction of any pathology for each examination into three classes: normal, uncertain, abnormal. Anomaly score: ratio of outlier voxels to entire brain ranging from 0 to 1 Anomaly heat map: voxels where value was outside the CIs
Ginat (2020) [23]	CT	ICH	Aidoc (see Buls 2021 above)		
Ginat (2021) [22]	CT	ICH			
Kuo (2019) [30]	CT	ICH	CNN, ‘PatchFCN’ (modified ResNet-38 architecture)	Manual segmentations for each examination	Binary prediction of ICH (present/not present) and lesion segmentations for each examination
Monteiro (2020) [28]	CT	ICH	CNN, DeepMedic architecture	Semi-automatically created segmentations for each examination	Binary prediction of ICH (present/not present) and lesion segmentations for each examination (output segmentations > 1 ml were considered as ICH present)
McLouth (2021) [26]	CT	ICH	Avicenna.ai, CINA v1.0: proprietary AI model	Not disclosed	Binary prediction of acute, hyperdense ICH (present/not present) for each examination
Prevedello (2017) [36]	CT	ICH, mass effect, hydrocephalus (‘algorithm 1’)	CNN, modified GoogLeNet architecture	Examination-level presence of abnormality (present/not present)	Binary prediction of pathology (present/not present) for each examination
Prevedello (2017) [36]	CT	Acute infarct (‘algorithm 2’)			
Salehinejad (2021) [27]	CT	ICH	CNN, ensemble model of modified ResNeXt-50 and ResNeXt-101 architectures, both pre-trained from ImageNet	Slice-level binary presence of abnormality (present/not present)	Binary prediction of ICH (present/not present) for each examination
Wang (2021) [29]	CT	ICH	Ensemble model of CNN and two recurrent neural networks. 1st place in the 2019-RSNA “Brain CT Hemorrhage Challenge”	Slice-level binary presence of abnormality (present/not present)	Binary prediction of ICH (present/not present) for each slice and examination
Voter (2021) [25]	CT	ICH	Aidoc (see Buls, 2021)		
Gauriau (2021) [35]	MR	Any pathology	CNN with 10 convolutional layers and one fully connected layer	Examination-level binary presence of abnormality (present/not present)	Binary prediction of pathology (present/not present) for each examination
Nael (2021) [32]	MR (FLAIR, ADC, DWI)	Any pathology	CNN, modified U‑net architecture	Examination-level binary presence of abnormality (present/not present)	Binary prediction of pathology (present/not present) for each examination
Nael (2021) [32]	MR (FLAIR, ADC, DWI)	ICH			
Nael (2021) [32]	MR (FLAIR, ADC, DWI)	Acute infarct			

Aidoc, Qure.ai and Avicenna.ai are commercial vendors of AI products. Aidoc v1.0, Aidoc v1.3 and CINA v1.0 are commercial AI solutions. Mask R‑CNN, PatchFCN, GoogLeNet, ResNet18, ResNet38, ResNeXt-50, ResNeXt-101, U‑net, DeepLab and DeepMedic are CNN architectures, published in academic literature. ImageNet is a large visual database often used in computer vision research; pretraining a model on ImageNet is a form of transfer learning

*CT* computed tomography, *MR* magnetic resonance, *FLAIR* fluid-attenuated inversion recovery, *DWI* diffusion weighted imaging, *ADC* apparent diffusion coefficient. FLAIR, DWI and ADC are commonly used MR sequences. *ICH* intracranial hemorrhage, *CNN* convolutional neural network, *RSNA* Radiological Society of North America

### Assessment of Risk of Bias

The risk of bias evaluation for each study using the QUADAS‑2 tool is summarised in Fig. 1. A high risk of bias in at least one domain was shown in 15/16 (94%) of studies. The modified signalling questions used for assessing each study, and their explanations are in Supplementary Materials 3 and 6, respectively.

The following were the commonest sources of bias: eight studies (8/16, 50%) assessed AI model performance in laboratory conditions only (“analytical validation” [21, 25, 26, 28–30, 33, 35, 37]). In contrast, four studies (4/16, 25%) placed the AI model within the clinical pathway (“clinical validation”) [22–24, 32], which more closely resembles a “real world” environment and therefore the intended applicability. Seven studies (7/16, 44%) used temporal validation alone, and therefore had a high risk of bias for patient selection as there is limited assessment of generalisability [26, 30–32, 34–36], compared to 9/16 (56%) studies where AI models were externally validated on test data from other institutions [21–25, 27–29, 33]. Studies that used fewer than two radiologists to assess the images of a scan for their reference standard were considered at high risk of bias, as individual radiologists do not have perfect accuracy—five studies (5/16, 31%) were therefore considered to have high risk of bias as only the clinical report was reviewed [22, 27, 32, 33, 36]. One study had a high risk of bias as the reference standard was informed by the output of the AI model (the index test) [25]; this study was therefore excluded from the meta-analysis.

### Analysis

The primary outcome for each study was diagnostic test accuracy, which is summarised in Table 2. Studies varied greatly in accuracy performance (sensitivity range: 0.70–1.00, specificity range: 0.51–1.00), but the highest accuracies were typically seen for intracranial hemorrhage detection using CT (Supplementary Fig. 2). Only two studies validated AI that used MRI; two AI models that detected any pathology using MRI had modest accuracies (sensitivity range: 0.78–1.00, specificity range: 0.65–0.80), compared to single pathology performance (Supplementary Fig. 3).

**Table 2:** Diagnostic test accuracy for included studies. Developers can choose different “operating points” which allows AI models to favour either sensitivity or specificity

Study	Modality, target	Training set (*n*)	Test set (*n*)	P (*n*, %)	Validation (test set separation, and if clinically validated)	TP	FN	FP	TN	ROC‑AUC (95 CI)	Sensitivity	Specificity	PPV	Prevalence adjusted-PPV
Chilamkurthy [21], Qure.ai, high sensitivity operating point	CT, ICH	4304 (165809 slices)	491 (CQ500 dataset)	205 (42%)	Geographical	194	11	83	203	0.942 (0.919−0.965)	0.946	0.710	0.700	0.266
Chilamkurthy [21], Qure.ai, high specificity operating point						168	37	30	256		0.820	0.895	0.848	0.465
Chilamkurthy [21], Radiologist comparators		N/A			N/A	201	4	29	257	N/A	0.980	0.899	0.874	0.518
						190	15	6	280		0.927	0.979	0.969	0.831
						187	18	6	280		0.912	0.979	0.969	0.829
Monteiro [28], high sensitivity operating point		655			Geographical	184	21	140	145	0.83 (0.79–0.87)	0.898	0.509	0.568	0.169
Monteiro [28], high specificity operating point						121	84	29	256		0.590	0.898	0.807	0.392
Wang [29], high sensitivity operating point		19530 (674258 slices)			Geographical	198	8	19	266	0.985 (0.977–0.993)	0.961	0.933	0.912	0.616
Wang [29], high specificity operating point						197	10	16	266		0.952	0.943	0.925	0.651
Kuo [30]		4396	200	25 (13%)	Temporal	25	0	23	152	0.991 (0.985–0.997)	1.00	0.87	0.521	0.458
Kuo [30], Radiologist comparators		N/A				24	1	5	170	N/A	0.96	0.97	0.828	0.789
						24	1	0	175		0.96	1.00	1.000	1.000
						16	9	4	171		0.63	0.98	0.800	0.757
						20	5	7	168		0.79	0.96	0.741	0.690
Ginat [23], Aidoc		~50000	2011	373 (19%)	Geographical, clinical	275	35	98	1603	–	0.887	0.942	0.737	0.631
Ginat [22], Aidoc			8723	1760 (20%)	Geographical, clinical	1555	205	274	6689	–	0.884	0.961	0.850	0.714
Buls [24], Aidoc			388	37 (10%)	Geographical, clinical	31	6	20	331	–	0.838	0.943	0.608	0.620
Voter [25], Aidoc			3605	349 (10%)	Geographical	322	27	74	3182	–	0.923	0.977	0.813	0.819
Arbabshirani [32]		24882	347	86 (25%)	Temporal, clinical	60	26	34	230	–	0.698	0.871	0.638	0.376
Chang [31]		10159	682	82 (12%)	Temporal	78	4	16	584	0.981	0.951	0.973	0.829	0.799
Salehinejad [27]		21784	5965	674 (11%)	Geographical	615	59	313	4978	0.954	0.912	0.941	0.663	0.819
McLouth [26], Avicenna.ai	CT, acute ICH	8994	814	255 (31%)	Temporal	233	22	14	545	–	0.914	0.975	0.943	0.802
Prevedello [36]	CT, ICH, mass effect, acute hydrocephalus	197	80	50 (63%)	Temporal	45	5	12	68	0.91	0.900	0.850	0.789	–
Prevedello [36]	CT, infarct	57	49	21 (43%)	Temporal	13	8	1	27	0.81	0.619	0.964	0.929	–
Chilamkurthy [21], Qure	CT, skull fracture	290055	491	39 (8%)	Geographical	37	2	61	391	0.962 (0.920–1.00)	0.949	0.865	0.378	–
Chilamkurthy [21], Qure	CT, mass effect	290055	491	115 (26%)	Geographical	115	12	23	104	0.922 (0.888–0.955)	0.906	0.819	0.833	–
Finck [34], ‘uncertain’ label considered abnormal	CT, any abnormality	191	248	178 (72%)	Temporal	178	0	44	26	–	1.000	0.371	0.802	–
Nael [33]	MR, any abnormality	9845	1072	867 (81%)	Geographical	691	175	41	164	0.88	0.80	0.80	0.94	–
	ICH	9845	1072	78 (7%)	Geographical	56	22	119	875	0.83	0.72	0.88	0.32	–
	Infarct	9845	1072	287 (27%)	Geographical	258	29	23	762	0.97	0.90	0.97	0.92	–
	Mass effect	9845	1072	31 (3%)	Geographical	25	6	198	843	0.87	0.81	0.81	0.12	–
Gauriau [35]	MR, any abnormality	2741	1489	960 (64%)	Temporal	742	218	188	346	0.80 (0.77–0.82)	0.773	0.648	0.798	–

Qure.ai, Aidoc and Avicenna.ai are commercial vendors for AI products

*P (positive)* number of examinations with target pathology in test dataset, *TP* true positives, *FN* false negatives, *FP* false positives, *TN* true negatives, *ROC-AUC* area under receiver operating characteristic curve, *PPV* positive predictive value, *ICH* intracranial hemorrhage, *CT* computed tomography

**Fig. 2:**
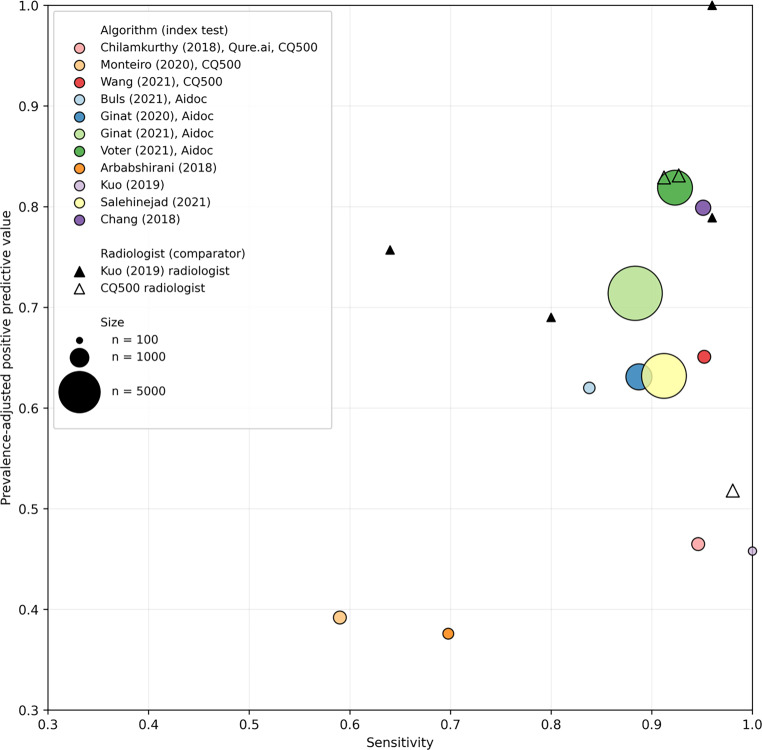
Diagnostic test accuracy of algorithms and comparators (single radiologists) in precision-recall space, for intracranial hemorrhage detection in CT imaging, when adjusted for an intracranial hemorrhage prevalence of 10%. The prevalence-adjusted positive predictive value (PPV) can be interpreted as the PPV you would expect for each model if the prevalence of ICH within the test dataset was 10%. An ideal classifier would be at the top-right corner at (1, 1). The size of each marker is proportional to the size of the test dataset. In studies where there was more than one operating point, we chose the operating point with the highest specificity. CQ500 = CQ500 external test dataset. Qure.ai, Aidoc and Avicenna.ai are commercial vendors for AI products

**Fig. 3:**
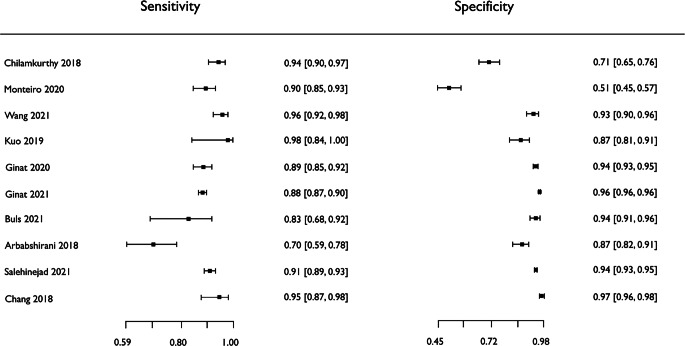
Forest plots demonstrating individual studies’ sensitivities and specificities

#### Diagnostic Test Accuracy of Radiologists Compared to AI

The performance of AI models against individual radiologists under laboratory conditions are available for four studies, summarised in Fig. 2. A full description of these studies can be found in Supplementary Material 7.

#### Clinical Implementation

All three (3/16, 19%) studies that also investigated clinical implementation performance, assessed the detection of intracranial hemorrhage using CT. Two (2/16, 13%) studies placed the AI model at the start of the clinical pathway before radiologist interpretation (pre-read triage) [22, 32], and 2/16 (13%) at the end after radiologist interpretation (post-read) [27, 32].

In the two studies where AI had been applied for pre-read triage, one showed a reduction in the time-to-report for non-urgent examinations which the AI flagged as abnormal, from a median time of 512 min to 19 min [32]. Radiologists in this study were unaware of the reprioritisation and were effectively blinded from the output of the AI. The other study was unblinded, as radiologists were made aware of examinations that the AI predicted to be abnormal [22]. This study demonstrated significant reductions in the mean time-to-report for flagged examinations for outpatients (674 to 70 min, *p* < 0.001), inpatients (390 to 352 min, *p* = 0.002), but not emergency cases (*p* = 0.37), or an undefined “other” class (*p* = 0.25) [22]. Importantly, neither study examined the extent and potential harms of delaying non-flagged studies, particularly AI false negatives which occurred in 26/347 (7.5%) [32] and 205/1760 (11.6%) [22] of outputs, respectively.

In two studies (2/16, 13%), AI had been applied as a second reader after radiologist interpretation and discrepancies between radiologists and AI were examined [27, 32]. AI was able to identify 4/347 (1.2%) and 2/5965 (0.03%) of intracranial hemorrhages that radiologists had missed (radiologist false negatives), respectively. If implemented, both studies estimated that the radiologist would be alerted that there was a discrepancy between them and the AI model in 10% (34/347) and 5% (313/5965) of cases, respectively, and 9 and 157 re-reviews would be required for 1 change in report, respectively [32]. In the second study, radiologist-positive and AI-negative discrepancies were also examined (59/5965, 1%) and found these were all AI false negatives—AI was unable to identify any radiologist overcalls (radiologist false positives). If AI were to be implemented to identify overcalls (radiologist false positives) as well as misses (radiologist false negatives), the radiologist would be alerted in 6% (372/5965) of cases and 186 re-reviews would be required for 1 change in a report [27].

#### Meta-analysis

Six (6/16, 38%) studies were unsuitable for meta-analysis. We excluded five studies (5/16, 31%) with heterogeneous modality or target conditions (one study detected hyperdense, intracranial hemorrhage only as might typically be seen in an acute setting, and did not consider isodense or hypodense intracranial hemorrhage as might typically be seen in a subacute or chronic setting) [26, 33–36]. One study (1/16, 6%) was excluded due to the fundamental methodological flaw of having a circular reference standard as described above [25]. The remaining subgroup of studies were those detecting intracranial hemorrhage using CT and applying CNNs, and consisted of 10 studies (10/16, 63%) [21–24, 27–32]; we included these studies for meta-analysis. Forest plots of sensitivity and specificity (Fig. 3) graphically showed a high level of heterogeneity. There was significant heterogeneity observed in both sensitivity and specificity; the χ^2^-test *p*-values were both < 0.001 and I^2^ statistics were 85.4% and 99.3%, respectively. The pooled sensitivity for intracranial hemorrhage detection in CT = 0.901 (95% confidence interval [CI] 0.853–0.935), and the pooled specificity = 0.903 (95% CI 0.826–0.948). The derived pooled measures of balanced accuracy = 0.931 (95% CI 0.889–0.957); positive likelihood ratio = 26.7 (95% CI 15.8–42.3); negative likelihood ratio = 0.106 (95% CI 0.0471–0.199); and diagnostic odds ratio = 280.0 (95% CI 128.0–533.0).

Heterogeneity was investigated using metaregression, which compared the pooled sensitivities and specificities of two subsets of the studies: three studies where different AI models were applied on the same test dataset (CQ500) [21, 28, 29], and three studies where the same AI model (Aidoc) was applied on different test datasets [22–24]. Using the Aidoc subset as a baseline, the CQ500 subset had higher pooled sensitivity (*p* = 0.008) and lower pooled specificity (*p* = 0.004), implying that AI model type and patient make-up in the test dataset contributed to the heterogeneity observed.

**Fig. 4:**
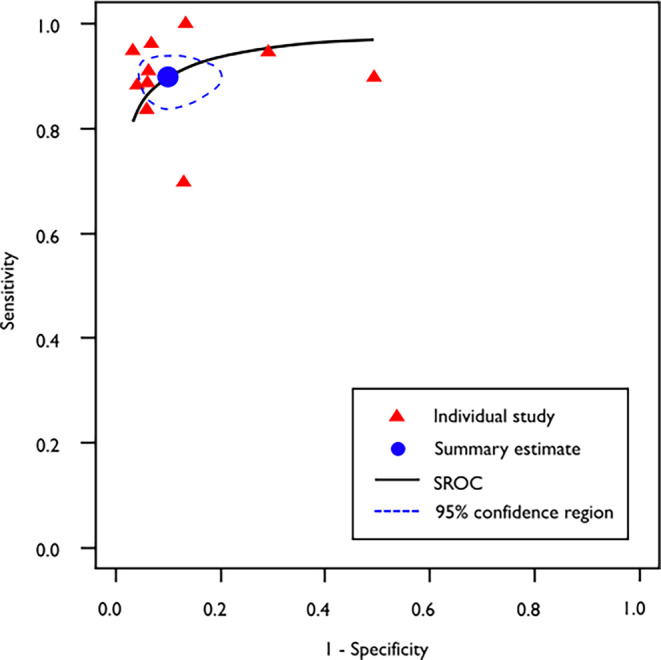
Summary receiver operating characteristic (SROC) curve for intracranial hemorrhage detection in CT imaging. A bivariate random effects model was used for meta-analysis, which allowed the estimation of the summary ROC (SROC) curve

Individual study ROC point estimates resulted in a summary ROC (SROC) curve (Fig. 4), for which the summary ROC-AUC = 0.948.

## Discussion

### Summary

This study aimed to determine the diagnostic accuracy of AI systems used to identify abnormalities in first-line neuroimaging tasks. Any productivity gains in such tasks are important as they are high volume and performed in almost all hospitals. To ensure our analyses were only focused on those studies that were not compromised by unrepresentative datasets or inadequate validation methodology, we excluded many studies (1239) that did not validate the AI model using datasets from representative clinical cohorts and many (218) for validating without temporal or external validation. Only sixteen studies were of sufficient rigour to be eligible for inclusion; however, even for these studies the overall methodological quality remained low with a high risk of bias in 94% of studies. Furthermore, most included studies were retrospective, with only four studies validating their AI models in clinical environments prospectively in real time (i.e. clinical validation).

For CT imaging studies, a subgroup of 10 AI models used to detect intracranial hemorrhage using CNNs, had a pooled sensitivity and specificity of 0.90, with a summary ROC-AUC of 0.95. Metaregression suggested that differences in the AI model development and patient selection contributed to the significant heterogeneity observed in both pooled measures (sensitivity I^2^ = 85.4%, specificity I^2^ = 99.3%). Four CT imaging studies allowed direct comparison between AI models with radiologists under laboratory conditions—further discussion is provided in Supplementary Material 7.

For MRI, only two studies were included. Both studies validated AI models that detect all pathologies. Together with a third study that used CT, a limitation of the three AI models that detect all pathologies is that findings seen in healthy ageing such as small vessel disease and age-commensurate atrophy are considered abnormal—this is reflected in the high prevalence of what was assigned as pathological in their test datasets (64–81%) [33–35]. AI that overcalls all older patients as abnormal raises concerns for applicability in clinical practice.

There were only three clinical implementation studies where AI was placed within the clinical pathway, as pre-read triage and for post-read discrepancy identification [22, 27, 32]. No study demonstrated a downstream clinical or health economic benefit.

### Strengths and Limitations

A strength of this study was that the search strategy was sensitive [38, 39]. This allowed the identification of a wide range of studies included in this review, many of which were missing in other systematic reviews for the general use of AI in neuroimaging [40–43]. We also included all AI methods, not just those limited to deep learning. Whilst broad inclusion is a study strength, it is also conceivable that summary performance accuracy might be diminished by the inclusion of older AI models; however, older AI models barely contributed to our results as 88% of all eligible studies, and 100% of studies in the meta-analysis subgroup used CNNs.

Another strength, unique to this study, was that the inclusion criteria were designed to only include studies where outcome metrics would have a reasonable chance of generalising to first-line neuroimaging in routine clinical practice. Therefore, the diagnostic test accuracies presented here are plausibly more generalisable than if less stringent inclusion criteria were used. Specifically, we first excluded studies that did not validate AI on temporally distinct or external test datasets. Second, we excluded studies that did not test on representative patient cohorts (which as a minimum standard required normal brains, the target condition and at least one non-target condition). As a result, we excluded studies that validated AI models on test datasets that contained the target condition and healthy controls only, which does not reflect the “real world”; we note that almost all ischemic stroke detection studies were therefore excluded [44].

A limitation is that meta-analysis was only suitable for one subgroup where there were sufficient homogeneous studies using the same imaging modality, target condition and AI model type. Another limitation is that no formal assessment of publication bias was undertaken; however, it is unlikely that our overall conclusions would change if studies with poorer AI model performances had been published.

### Strategies for Implementing AI into Clinical Pathways

The standalone diagnostic accuracy of AI to detect abnormalities has been demonstrated to be high, particularly for intracranial hemorrhage in CT imaging. There was insufficient evidence, however, to suggest where such AI would be most useful in the clinical pathway. This included those being marketed commercially (Supplementary Material 8).

Both studies that investigated intracranial hemorrhage AI detectors for pre-read CT worklist triage found that the greatest reduction in reporting time was for outpatient examinations when compared to emergency or inpatient examinations [22, 32]. There was insufficient evidence, however, from these and any other studies regarding the downstream clinical benefit and cost-effectiveness of AI implementation. AI models with poor sensitivity in a pre-read triage setting would systemically increase the time to report AI false negative examinations as these would be put at the back of the reporting queue; it was unclear from both studies whether AI false negatives were significantly delayed and the extent of harm, if any, that was associated with this delay. For pre-read triage, it is also unknown whether knowing that AI puts flagged examinations to the front of the queue could have long-term consequences on radiologist performance. There is a similar question regarding AI intended as a second reader which may unintentionally affect the behaviour of radiologists; for example, the implementation of automated computer-assisted diagnosis (CAD) tools in mammography, to be used as a reader aid during radiologist interpretation, has previously been shown to reduce radiologist sensitivity [45] and overall accuracy [46].

One advantage of a post-read implementation is that radiologists are initially blinded to the AI decision. In a post-read setting, AI models could be used to flag discrepancies to determine potential radiologist “misses” or “overcalls” and allow a re-review. An AI model with poor specificity or PPV in this setting would create a high burden on radiologist time with a large number of false positive scans to re-review. In the two studies that investigated discrepancies, there appeared to be low additive diagnostic yield associated with a high rate of re-review. Therefore, further studies will be necessary to understand the cost-effectiveness of such post-read strategies.

Many AI models developed for high-sensitivity pre-read triage could plausibly be repurposed as high-PPV post-read discrepancy identifiers and vice versa simply by adjusting the operating threshold.; however, for any specific downstream clinical task it is necessary to further validate any predictive model following such recalibration.

## Conclusion

We have analysed the evidence and presented the diagnostic performance of the current state-of-the-art AI detection models that can be applied to first-line neuroimaging. Such tasks are important as they are high volume and performed in almost all hospitals and offer considerable potential for the necessary productivity gains required in the twenty-first century. If the intended use of AI detection models is as a tool to improve radiologist efficiency rather than a replacement for radiologists, AI may be clinically useful even if the accuracy shown in our meta-analysis remains lower than that of radiologists for the task of intracranial hemorrhage detection; however, at present, there is insufficient evidence to recommend implementation of AI for abnormality detection, including hemorrhage detection, into any part of the clinical pathway. Importantly, the clinical and health economic benefits are currently unproven. For now, future research efforts should aim to minimise bias and demonstrate analytical validation through well-designed studies using clinically representative external test datasets which can unequivocally prove high performance accuracy and good generalisability. Following this, clinical trials will be required to confirm the performance findings in the “real world” and determine whether the clinical benefits of implementing AI in the clinical pathway outweigh the potential harm to patients. In addition to clinical validation, such trials could include health economic analyses to determine the costs incurred and benefits obtained within the wider healthcare system.

### Supplementary Information

Supplementary Material: A systematic review of artificial intelligence for abnormality detection in high volume neuroimaging and sub-group meta-analysis for intracranial hemorrhage detection
