# Supplementary material for: Systematic Review of Artificial Intelligence for Abnormality Detection in High-volume Neuroimaging and Subgroup Meta-analysis for Intracranial Hemorrhage Detection
Source: Clin Neuroradiol. 2023 Jun 1;33(4):943–56. doi: 10.1007/s00062-023-01291-1 (PMC10233528; doi:10.1007/s00062-023-01291-1)
Supplement: Supplementary file 1 — Supplementary Material: A systematic review of artificial intelligence for abnormality detection in high volume neuroimaging and sub-group meta-analysis for intracranial hemorrhage detection [file 62_2023_1291_MOESM1_ESM.docx]

**Supplementary Material: A systematic review of artificial intelligence for abnormality detection in high volume neuroimaging and sub-group meta-analysis for intracranial hemorrhage detection**

Contents

[Supplementary Material 1: Search strategy 2](#_Toc125929385)

[Supplementary Material 2: Full data extraction and data analysis strategy 5](#_Toc125929386)

[Inclusion criteria 5](#_Toc125929387)

[Exclusion criteria 5](#_Toc125929388)

[Study selection 5](#_Toc125929389)

[Data extraction and quality assessment 6](#_Toc125929390)

[Data analysis 6](#_Toc125929391)

[Meta-analysis 6](#_Toc125929392)

[Supplementary Material 3: Modified QUADAS-2 signalling questions 7](#_Toc125929393)

[Supplementary Material 4: Calculations for prevalence adjusted PPV, confusion matrices from provided accuracy metrics 9](#_Toc125929394)

[Supplementary Material 5: Summary of characteristics for each study. 10](#_Toc125929395)

[Supplementary Material 6: Further details for the assessment of risk of bias 14](#_Toc125929396)

[Patient Selection 14](#_Toc125929397)

[Index test 14](#_Toc125929398)

[Reference Standard 14](#_Toc125929399)

[Flow and timing 14](#_Toc125929400)

[Supplementary Material 7: Direct comparison to radiologists 15](#_Toc125929401)

[Supplementary material 8: Commercial solutions 16](#_Toc125929402)

[Supplementary Figures 17](#_Toc125929403)

[References 20](#_Toc125929404)

# Supplementary Material 1: Search strategy

| MEDLINE (OVID)  1. exp Magnetic Resonance Imaging/  2. (mri or mr or (magnetic adj1 resonance)).ab,ti.  3. exp Tomography, X-Ray Computed/  4. (ct or (comput* adj1 tomograph*)).ab,ti.  5. (cat adj3 (scan* or imag* or stud*)).ab,ti.  6. neuroimag*.ti,ab.  7. 1 or 2 or 3 or 4 or 5 or 6  8. (brain or head or skull or cerebral or intracerebral or cerebrum or cranial or intracranial or cranium).ab,ti.  9. (detect* or classif* or identif* or diagnos* or predict* or decis* or decid*).ti,ab.  10. 7 and 8 and 9  11. exp Diagnosis, Computer-Assisted/ or exp Algorithms/ or exp Artificial Intelligence/ or exp Machine Learning/ or exp Neural Networks, Computer/ or exp Pattern Recognition, Automated/  12. ((artificial adj1 intelligence) or ((deep or machine) adj1 learning)).mp.  13. algorithm*.ab,ti,kw,kf. or automat*.ab,ti. or radiomic*.mp. or (comput* adj3 (aid* or assist* or vision*)).ti,ab.  14. ((supervised or unsupervised or (semi adj1 supervised) or deep hybrid or cluster* or bayes* or gauss*) adj3 (learning or model* or net* or algo*)).mp.  15. ((feature adj3 (engineer* or select* or extract* or learn*)) or hyperparameter).mp. [mp=title, abstract, original title, name of substance word, subject heading word, floating sub-heading word, keyword heading word, organism supplementary concept word, protocol supplementary concept word, rare disease supplementary concept word, unique identifier, synonyms]  16. (((neural or conv*) adj1 (net* or learn* or model*)) or CNN or convnet or RNN, or long short-term memory or lstm or gate* recurrent unit or gru or boltzmann machine or deep belief net* or spatial transformer net* or sum product network).mp.  17. ((ensemble or transfer or zero shot or reinforcement or dictionary) adj1 (learning or model* or net* or algo*)).mp.  18. (vector machine or SVM or ((classification or regression or probability or decision) adj1 tree*) or random forest).mp.  19. (generative model* or autoencod* or aae or vae or cae or dae or sdae or gan or generative adversarial).mp.  20. (pca or principal component analysis or (k adj1 means) or (nearest adj1 neighbo?r) or knn or (fuzzy adj3 logi*) or isolation forest or hidden markov model or association rule* or feature bag* or score normali$ation).mp.  21. 11 or 12 or 13 or 14 or 15 or 16 or 17 or 18 or 19 or 20  22. 10 and 21 |
| --- |
| EMBASE (OVID)  1. exp nuclear magnetic resonance imaging/  2. (mri or mr or (magnetic adj1 resonance)).ab,ti.  3. computer assisted tomography/  4. (ct or (comput* adj1 tomograph*)).ab,ti.  5. (cat adj3 (scan* or imag* or stud*)).ab,ti.  6. neuroimag*.ti,ab.  7. 1 or 2 or 3 or 4 or 5 or 6  8. (brain or head or skull or cerebral or intracerebral or cerebrum or cranial or intracranial or cranium).ab,ti.  9. (detect* or classif* or identif* or diagnos* or predict* or decision or decis* or decid*).ti,ab.  10. 7 and 8 and 9  11. computer assisted diagnosis/ or exp algorithm/ or exp artificial intelligence/ or exp machine learning/  12. ((artificial adj1 intelligence) or ((deep or machine) adj1 learning)).mp.  13. (algorithm* or automat*).ab,ti. or radiomic*.mp. or (comput* adj3 (aid* or assist* or vision*)).ti,ab.  14. ((supervised or unsupervised or (semi adj1 supervised) or deep hybrid or cluster* or bayes* or gauss*) adj3 (learning or model* or net* or algo*)).mp.  15. ((feature adj3 (engineer* or select* or extract* or learn*)) or hyperparameter).mp.  16. (((neural or conv*) adj1 (net* or learn* or model*)) or CNN or convnet or RNN, or long short-term memory or lstm or gate* recurrent unit or gru or boltzmann machine or deep belief net* or spatial transformer net* or sum product network).mp.  17. ((ensemble or transfer or zero shot or reinforcement or dictionary) adj1 (learning or model* or net* or algo*)).mp.  18. (vector machine or SVM or ((classification or regression or probability or decision) adj1 tree*) or random forest).mp.  19. (generative model* or autoencod* or aae or vae or cae or dae or sdae or gan or generative adversarial).mp.  20. (pca or principal component analysis or (k adj1 means) or (nearest adj1 neighbo?r) or knn or (fuzzy adj3 logi*) or isolation forest or hidden markov model or association rule* or feature bag* or score normali$ation).mp.  21. 11 or 12 or 13 or 14 or 15 or 16 or 17 or 18 or 19 or 20  22. 10 and 21 |
| Web of Science   1. TI =(mri or mr or (magnetic NEAR/0 resonance) ) OR AB=(mri or mr or (magnetic NEAR/0 resonance) ) 2. TI=(ct or (comput* NEAR/0 tomograph*) ) OR AB=(ct or (comput* NEAR/0 tomograph*) ) 3. TI=(cat NEAR/2 (scan* or imag* or stud*) ) OR AB=(cat NEAR/2 (scan* or imag* or stud*) ) 4. TI=(neuroimag*) OR AB=(neuroimag*) 5. #4 OR #3 OR #2 OR #1 6. TI=(brain or head or skull or cerebral or intracerebral or cerebrum or cranial or intracranial or cranium) OR AB=(brain or head or skull or cerebral or intracerebral or cerebrum or cranial or intracranial or cranium) 7. TI=(detect* or classif* or identif* or diagnos* or predict* or decision or decid*) OR AB=(detect* or classif* or identif* or diagnos* or predict* or decision or decid*) 8. #7 AND #6 AND #5 9. TI=((artificial NEAR/0 intelligence) or ((deep or machine) NEAR/0 learning)) OR AB=((artificial NEAR/0 intelligence) or ((deep or machine) NEAR/0 learning)) 10. TI=(algorithm* or automat* or radiomic* or (comput* NEAR/2 (aid* or assist* or vision*) )) OR AB=(algorithm* or automat* or radiomic* or (comput* NEAR/2 (aid* or assist* or vision*) )) 11. TI = (supervised NEAR/2 (learning or model* or net* or algo*) ) OR AB = (supervised NEAR/2 (learning or model* or net* or algo*) ) 12. TI = (unsupervised NEAR/2 (learning or model* or net* or algo*) ) OR AB= (unsupervised NEAR/2 (learning or model* or net* or algo*) ) 13. TI=(semi supervised NEAR/2 (learning or model* or net* or algo*) ) OR AB=(semi supervised NEAR/2 (learning or model* or net* or algo*) ) 14. TI=(deep hybrid NEAR/2 (learning or model* or net* or algo*) ) OR AB=(deep hybrid NEAR/2 (learning or model* or net* or algo*) ) 15. TI=(bayes* NEAR/2 (learning or model* or net* or algo*) ) OR AB=(bayes* NEAR/2 (learning or model* or net* or algo*) ) 16. TI=(cluster* NEAR/2 (learning or model* or net* or algo*) ) OR AB=(cluster* NEAR/2 (learning or model* or net* or algo*) ) 17. TI=(gauss* NEAR/2 (learning or model* or net* or algo*) ) OR AB=(gauss* NEAR/2 (learning or model* or net* or algo*) ) 18. TI=((feature NEAR/2 (engineer* or select* or extract* or learn*) ) or hyperparameter) OR AB=((feature NEAR/2 (engineer* or select* or extract* or learn*) ) or hyperparameter) 19. TI=(((neural or conv*) NEAR/0 (net* or learn* or model*) ) or CNN or convnet or RNN, or long short-term memory or lstm or gate* recurrent unit or gru or boltzmann machine or deep belief net* or spatial transformer net* or sum product network) OR AB=(((neural or conv*) NEAR/0 (net* or learn* or model*) ) or CNN or convnet or RNN, or long short-term memory or lstm or gate* recurrent unit or gru or boltzmann machine or deep belief net* or spatial transformer net* or sum product network) 20. TI=(ensemble NEAR/0 (learning or model* or net* or algo*) ) OR AB=(ensemble NEAR/0 (learning or model* or net* or algo*) ) 21. TI=(transfer NEAR/0 (learning or model* or net* or algo*) ) OR AB=(transfer NEAR/0 (learning or model* or net* or algo*) ) 22. TI=(zero shot NEAR/0 (learning or model* or net* or algo*) ) OR AB=(zero shot NEAR/0 (learning or model* or net* or algo*) ) 23. TI=(reinforcement NEAR/0 (learning or model* or net* or algo*) ) OR AB=(reinforcement NEAR/0 (learning or model* or net* or algo*) ) 24. TI=(dictionary NEAR/0 (learning or model* or net* or algo*) ) OR AB=(dictionary NEAR/0 (learning or model* or net* or algo*) ) 25. TI=((vector machine or SVM or ((classification or regression or probability or decision) NEAR/0 tree*) or random forest)) OR AB=((logistic regression or vector machine or SVM or ((classification or regression or probability or decision) NEAR/0 tree*) or random forest)) 26. TI=((generative model* or autoencod* or aae or vae or cae or dae or sdae or gan or generative adversarial)) OR AB=((generative model* or autoencod* or aae or vae or cae or dae or sdae or gan or generative adversarial)) 27. TI=(pca or principal component analysis or (k near/0 means) or (nearest near/0 neighbo$r) or knn or (fuzzy near/0 logi*) or isolation forest or hidden markov model or association rule* or feature bag* or score normali$ation) OR AB=(pca or principal component analysis or (k near/0 means) or (nearest near/0 neighbo$r) or knn or (fuzzy near/0 logi*) or isolation forest or hidden markov model or association rule* or feature bag* or score normali$ation) 28. #27 OR #26 OR #25 OR #24 OR #23 OR #22 OR #21 OR #20 OR #19 OR #18 OR #17 OR #16 OR #15 OR #14 OR #13 OR #12 OR #11 OR #10 OR #9 29. #28 AND #8 |
| Cochrane library  ID Search  #1 MeSH descriptor: [Magnetic Resonance Imaging] explode all trees  #2 (mri or mr or (magnetic adj1 resonance)):ab,ti  #3 MeSH descriptor: [Tomography, X-Ray Computed] explode all trees  #4 (ct or (comput* adj1 tomograph*)):ab,ti  #5 (cat adj3 (scan* or imag* or stud*)):ab,ti  #6 neuroimag*:ti,ab  #7 {OR #1-#6}  #8 (brain or head or skull or cerebral or intracerebral or cerebrum or cranial or intracranial or cranium):ti,ab  #9 (detect* or classif* or identif* or diagnos* or predict* or decision or decis* or decid*):ti,ab  #10 {AND #7-#9}  #11 MeSH descriptor: [Diagnosis, Computer-Assisted] explode all trees  #12 MeSH descriptor: [Algorithms] explode all trees  #13 MeSH descriptor: [Artificial Intelligence] explode all trees  #14 MeSH descriptor: [Neural Networks, Computer] explode all trees  #15 MeSH descriptor: [Machine Learning] explode all trees  #16 ((artificial adj1 intelligence) or ((deep or machine) adj1 learning))  #17 algorithm*:ab,ti,kw or automat*:ab,ti or radiomic*:ab,ti,kw or (comput* adj3 (aid* or assist* or vision*)):ab,ti  #18 ((supervised or unsupervised or (semi adj1 supervised) or deep hybrid or cluster* or bayes* or gauss*) adj3 (learning or model* or net* or algo*))  #19 ((feature adj3 (engineer* or select* or extract* or learn*)) or hyperparameter)  #20 (((neural or conv*) adj1 (net* or learn* or model*)) or CNN or convnet or RNN, or long short-term memory or lstm or gate* recurrent unit or gru or boltzmann machine or deep belief net* or spatial transformer net* or sum product network)  #21 ((ensemble or transfer or zero shot or reinforcement or dictionary) adj1 (learning or model* or net* or algo*))  #22 (vector machine or SVM or ((classification or regression or probability or decision) adj1 tree*) or random forest)  #23 (generative model* or autoencod* or aae or vae or cae or dae or sdae or gan or generative adversarial)  #24 (pca or principal component analysis or (k adj1 means) or (nearest adj1 neighbo?r) or knn or (fuzzy adj3 logi*) or isolation forest or hidden markov model or association rule* or feature bag* or score normali$ation)  #25 {OR #11-#24}  #26 #10 AND #25 |

# Supplementary Material 2: Full data extraction and data analysis strategy

## Inclusion criteria

We included studies where an AI model could predict if a given CT or MRI examination was abnormal. To prevent major methodological bias related to generalisability, eligible studies included were those validating the AI model on a test dataset separated from the dataset used for development, either by time as a minimum acceptable inclusion standard (temporal validation), or by location (geographical validation; defined as external validation in this review).

In routine clinical practice, imaging patient cohorts contain normal brains and a range of pathological conditions. For AI studies detecting a single pathological target condition such as intracranial hemorrhage, a representative study would require that the AI model was validated not only on cohorts that contained the target condition, but also a reasonably representative range of non-target conditions that might be obtained during routine scanning. As a minimum acceptable inclusion standard, eligible studies were required to have test datasets that contained normal scans, scans containing the target, and one or more non-target conditions.

## Exclusion criteria

The motivation for the study was to review abnormality detection in first-line, clinical neuroimaging.

Angiography or perfusion studies, for example, were excluded on the grounds that they would typically be used as second-line investigations triggered by a high clinico-radiological suspicion for a particular pathology (e.g., CT angiography in patients with clinico-radiological features of stroke caused by a large vessel occlusion). For the same rationale, other advanced MR techniques (e.g., MR spectroscopy) were excluded.

Target conditions with a structural correlate often seen at the individual level (e.g. Alzheimer’s disease) were included. In contrast, those conditions where structural differences have been shown only in group-wise comparison studies but are not apparent to the radiologist at the individual level, were excluded (e.g., a range of psychiatric conditions where voxel-based morphometry has shown structural differences when compared to healthy controls).

All studies using other forms of internal validation not adhering to the minimum acceptable inclusion standard of temporal validation, were excluded if there was no external validation. An example of this scenario is studies using cross-validation alone. Studies that only reported the accuracy of the AI model to make voxel-wise (e.g., segmentation studies) or slice-wise predictions but did not subsequently report at the patient level were excluded (unless patient level accuracy could be calculated from the published study data).

Studies testing exclusively on pediatric populations were excluded. Studies not published in a peer-reviewed journal or without an English language translation were excluded^1^.

## Study selection

S.A. and D.W. (radiologist and data scientist, both with 3 years neuroimaging research experience), independently reviewed the titles and abstracts of all retrieved records using the inclusion criteria. Full texts were then assessed for eligibility with any final arbitration through a third reviewer (T.C.B., neuroradiologist, 16 years neuroimaging research experience).

## Data extraction and quality assessment

Study quality was assessed independently (S.A. and D.W.) with any final arbitration through a third assessor (T.C.B.). We used the QUality Assessment of Diagnostic Accuracy Studies-2 (QUADAS-2) tool^2^, tailored to the review question incorporating items from the Checklist for Artificial Intelligence in Medical Imaging (CLAIM)^3^; modified signalling questions are presented in Supplementary Material 3.

# Supplementary Material 3: Modified QUADAS-2 signalling questions

| Question | Points specific to this review |
| --- | --- |
| **PARTICIPANT SELECTION – A. RISK OF BIAS** | |
| Was a consecutive or random sample of patients enrolled in the test set? | Yes: datasets of unenriched, consecutive patients OR randomly selected patients  Unclear: Not stated  No: Other |
| Did the study avoid inappropriate exclusions and does the study likely have a range of pathology matching a clinical cohort? | Yes: Clinical cohorts with no inappropriate exclusions  Unclear: Not stated  No: 3 or fewer categories of pathology |
| Was the test dataset geographically distinct from the training dataset | Yes: Geographically distinct  Unclear: Not stated  No: Temporally distinct only OR Internal validation only: e.g. hold-out, cross-validation |
| **PARTICIPANT SELECTION - B. CONCERNS REGARDING APPLICABILITY** | |
| Is there a concern that the included patients do not match the review question? | High concern if any of:   - A consecutive or random sample of patients weren’t used - An enriched sample not matching the prevalence of disease - 3 or fewer categories of pathology - Any pathology or subtype of pathology excluded |
| **INDEX TESTS – A. RISK OF BIAS** | |
| Were patients present in the test dataset not used for development? | Yes: The same patients are not in both test and training datasets  No: Evidence of data leakage |
| If a threshold was used, was it pre-specified? | Yes: Threshold set previously e.g. in a commercially available AI system, or threshold or operating point set during training  Unclear: Not stated  No: Accuracy metrics provided directly on the test dataset, without pre-specifying operating point during model development |
| **INDEX TESTS - B. CONCERNS REGARDING APPLICABILITY** | |
| Are there concerns that the index test, its conduct, or interpretation differ from the review question? | High concern if any of:  Analytical validation only (in laboratory conditions/analytical validation)  Low concern if:  AI deployed in clinical practice (clinical validation) |
| **REFERENCE STANDARD – A. RISK OF BIAS** | |
| Is the reference standard likely to correctly classify the target condition? | Yes if any of:   - 2 or more radiologists independently reviewed the images in a study, for each study in the test set. If labels were derived from existing radiology reports by 1 radiologist, a different radiologist must have reviewed the images. - Subsequent information available from different modality or histopathology e.g. DWI-MR providing the labels for ischemic stroke in CT   No: Fewer than 2 radiologists reviewed images for each study (e.g. 1 radiologist extracted labels from existing reports) |
| Were the reference standard results interpreted without knowledge of the results of the index test? | Yes: AI output was not known to individual(s) creating the reference standard  No: Reference standard informed by AI output |
| **REFERENCE STANDARD - B. CONCERNS REGARDING APPLICABILITY** | |
| Are there concerns that the target condition as defined by the reference standard does not match the review question? | High concern if any of:   - Labels in the test dataset were not generated by a radiologist (e.g. not a natural language processing model) - Findings expected in healthy ageing (e.g. cerebral atrophy and small vessel disease commensurate for age) are considered abnormal by the reference standard |
| **FLOW AND TIMING – A. RISK OF BIAS** | |
| Were all patients included in the analysis? | Yes: Patients in final analysis the same as in the initial sample.  No: Differing number of patients between sampling and results without adequate explanation. |

# Supplementary Material 4: Calculations for prevalence adjusted PPV, confusion matrices from provided accuracy metrics

The positive predictive value (PPV) is preferred to specificity in abnormality detection, as it is a measure of how well the target condition is correctly identified from all scans predicted to be positive by an AI model (true positives and false positives). Comparatively, specificity focuses on how well the normal class is identified from all scans that are negative for the target condition (true negatives and false positives). In extremely imbalanced datasets where the normal class has a far greater prevalence than the target condition, an AI model that classifies all data as ‘normal’ would have reasonable specificity, but poor PPV. PPV, however, is dependent on prevalence, therefore a prevalence-adjusted PPV needs to be calculated to compare AI models detecting the same target condition.

- 1. Calculating prevalence-adjusted positive predictive value (PPV) from sensitivity and specificity^11^:

$$PPV= \frac{sensitivity\cdot prevalence}{(1-specificity)(1-prevalence)+\left( sensitivity\cdot prevalence \right)}$$

If the prevalence of the target condition is consistently 10% across the compared studies, the prevalence-adjusted PPV can be calculated as follows:

- 1. Calculating the number of true positives (TP), false negatives (FN), false positives (FP), true negatives (TN) from sensitivity, specificity, the total number of scans (total) and the number of scans with the target condition in the test dataset (P):
  $N=total-P$
  $TP=sensitivity\cdot P$
  $TN=specificity\cdot N$
  $FN=P-TP$
  $FP=N-TN$
  2. Calculating the number of true positives (TP), false negatives (FN), false positives (FP), true negatives (TN) from sensitivity, specificity, the PPV the total number of scans in the test dataset (total):

$$TP=\frac{total}{\left( \frac{1}{PPV}-1 \right)\cdot\frac{specificity}{1-specificity}+\frac{1}{sensitivity}+\frac{1}{PPV}-1}$$

$$FN=TP\cdot\left( \frac{1}{sensitivity-1} \right)$$

$$FP=\frac{TP}{PPV}-TP$$

$$TN=TP\cdot\left( \frac{1}{PPV}-1 \right)\cdot\frac{specificity}{1-specificity}$$

# Supplementary Material 5: Summary of characteristics for each study.

| Study (author, year) | Modality, Target pathology | Index test | Training set | Test set | Reference standard (Ground truth for test set) |
| --- | --- | --- | --- | --- | --- |
| Arbabshirani (2018) | CT, ICH | CNN  Labels: Examination-level Output: Binary prediction of ICH (present/not present) for each examination | 46,583 CT head examinations from 31,256 patients, from 2007-2017, from 1 institution (USA), 4 scanner vendors, randomly split into training (24,882) and internal hold out test sets (6,374). Training set labels extracted from clinical reports. | 347 non-urgent inpatient and outpatient CT head studies during a 3 month implementation in clinical practice in 2017. | 25% of radiologist reports were converted to labels by research assistants under supervision of one neuroradiologist (>11,600), 75% using an NLP algorithm. |
| Buls (2021) | CT, ICH | Aidoc v1.3, a proprietary CNN  Labels: examination-level, bounding boxes, segmentation Output: Binary prediction of ICH (present/not present) for each examination, key images for review | Approximately 50,000 CT head examinations from 9 different sites, 17 scanner models. Ground truth labels varied depending on hemorrhage type and size, including study-level binary labels, slice-level bounding boxes and voxel-level segmentation. | A subset of 388 out of 500 CT head studies consecutively acquired from Sep - Oct 2019 at 1 institution in Brussels (Belgium), 4 scanners, 3 scanner vendors, with patients <18 years of age excluded. 112 studies were not done because the AI model could not process them in real-time, although the cause of failure was not investigated. | Consensus opinion for each study derived by three neuroradiologists with between 5 - 15 years of experience, with access to previous studies, clinical history and clinical reports. |
| Chang (2018) | CT, ICH | CNN, modified mask R-CNN architecture  Labels: examination-level, segmentation  Outputs: Binary prediction of ICH (present/not present) for each examination, segmentation and volume estimation of ICH | 10,159 CT head examinations, from 1 institution from January to August 2017. Examination-level labels created manually. | All 682 emergency department CT head examinations, from the same institution, consecutively acquired in February 2018. | ICH positive cases were identified from clinical reports and confirmed visually by a radiologist. Segmentation masks were generated semi-automatically by a radiologist. |
| Chilamkurthy (2018) | CT, ICH  CT, mass effect | Qure.ai proprietary CNN, modified ResNet18 architecture Labels: slice-level Output: Confidence score for each slice in examination, separate random forest machine learning model used to convert confidence for each slice into binary prediction of ICH for each examination | 313,318 CT head examinations from 2011-2017, collected from 20 institutions (India), 3 scanner vendors, 12 scanner models, randomly split into training (290,055) and internal hold out test sets (21,095). Slice-level labels were manually created for ICH (4304 scans, 165809 slices), midline shift and mass effect (699 scans, 26135 slices).  Bounding-box labels were manually created for fracture (1119 scans, 9938 slices) | "CQ500 dataset": Enriched dataset of 491 studies from 2012-2018, from 6 institutions in New Dehli (India), 2 scanner vendors, 6 scanner models, with postoperative patients and patients <7 years of age excluded. 214 scans consecutively collected in Nov 2017, 277 scans selected for ICH present in the report using an NLP tool. | Three radiologists independently recorded the presence of ICH, midline shift and fracture for all 491 studies, with majority vote deciding the labels for each study. |
| Chilamkurthy (2018) | CT, Skull fracture | Qure.ai proprietary CNN, modified DeepLab architecture  Labels: Bounding-box annotations per slice Output: Confidence score for each slice in examination, separate random forest model used to convert into binary prediction of fracture for each examination |  |  |  |
| Finck (2021) | CT, any pathology | “Weakly supervised machine learning”: normative learning by registering normal brains to a shared atlas and determining per-voxel confidence-intervals Labels: N/A - only trained on normal brains Output: Anomaly heat map: voxels where value was outside the CIs, Anomaly score: ratio of outlier voxels to entire brain ranging from 0 to 1, Prediction of any pathology for each examination into three classes: normal, uncertain, abnormal | 191 normal CT head examinations, from 2018 - Feb 2020, 1 institution (Germany), 1 scanner model for training. 31 pathological and 30 normal CT head examinations, 2018 - 2019, for validation. Examination-level labels assigned. | 248 consecutive CT head studies, Mar 2020, 1 institution, 1 scanner model, after excluding follow up examinations (170) and patients with metal implants (56). | Two neuroradiologists reviewed the images and report for each study to extract an examination-level label |
| Ginat (2020) | CT, ICH | Aidoc v1.3 (see Buls 2018 above) | Aidoc v1.3 (see Buls 2018 above) | 2,011 consecutively acquired urgent CT head studies, Jan - Feb 2019, from 1 tertiary hospital, 9 scanners | One neuroradiologist reviewed the images and clinical report for each study |
| Ginat (2021) | CT, ICH | Aidoc (see Buls 2018 above) | Aidoc (see Buls 2018 above) | 8,723 consecutively acquired CT head studies, May 2020 - Feb 2021, from 1 tertiary hospital, 9 scanners | ‘The final radiologist report defined the ground truth’ – implied that same radiologist that reported extracted the binary examination-level label |
| Kuo (2019) | CT, ICH | CNN, 'PatchFCN' (modified ResNet-38 architecture) Labels: Lesion segmentations Output: Lesion segmentations, prediction of ICH (present/not present) for each examination | 4,396 CT head studies from 2010-2017 of which 1,131 were positive for ICH, collected from affiliated hospitals of UCSF (USA), 2 scanner vendors, trained using randomly split 4-fold cross validation. Voxel-wise ICH segmentations, manually performed, used as labels for 1,131 positive scans. Studies required 'skull-stripping' as a pre-processing step. | 200 CT head studies performed at the same hospitals, November - December 2017. Excluding previous neurosurgery, from all CT head scans performed, 150 were randomly chosen if no previous studies were performed in the same visit, 50 were randomly chosen if more than one CT head was performed on the same visit. Skull-stripping failed on 1 study, which was replaced by another from the same time period. | Consensus opinion of two neuroradiologists for each examination |
| Monteiro (2020) | CT, ICH | CNN, DeepMedic architecture Labels: Lesion segmentations Output: Lesion segmentations, binary prediction of ICH (present/not present) for each examination created by only considering segmentations >1ml as ICH | 839 CT head studies from 512 patients, from 2014 - 2017, 60 institutions across Europe, subset of CENTER-TBI study, randomly split into training (184) and internal hold out test sets (655). Segmentation labels semi-automatically generated by manually correcting outputs of an earlier version of the AI model. | CQ500 (see Chilamkurthy, 2018) | CQ500 (see Chilamkurthy, 2018) |
| McLouth (2021) | CT, ICH | Avicenna.ai, CINA v1.0: proprietary AI model  Labels: not disclosed Output: Binary prediction of acute, hyperdense ICH (present/not present) for each examination | 8,994 CT head studies, multiple institutions through vRAD, a teleradiology service (USA), 2014-2018. The AI model 'was only trained to identify acute blood based off of hyperdense components', 'chronic hemorrhages cannot be identified by the AI model unless they contain more acute hyperdense components' | Patients were pre-selected for suspected ICH by search terms in the clinical indication e.g. 'hemorrhage', 'NCCT', 'head'. 395 CT head studies from vRAD (USA), 2019, 419 studies from University of California, Irvine (USA), 2017-2019, although sampling method was unclear. 4 scanner manufacturers. 10 studies excluded for insufficient image quality or contrast-enhanced. | Consensus of two neuroradiologists, with a third for arbitration. |
| Prevedello (2017) | CT, ICH, mass effect, hydrocephalus (‘algorithm 1’) | CNN, modified GoogLeNet architecture Labels: Examination-level Output: Binary prediction of pathology (present/not present) for each examination | 246 CT head studies, 1 institution (USA), >1 scanner model, 146 positive findings from a consecutive sample and 100 healthy controls from a random sample, from which 2583 2-D images with the representative pathology were split randomly into training (80%) and internal hold out test sets. Labels were extracted manually from clinical report. | 130 CT head studies from a consecutive sample of 226 studies in 2015, after exclusion of postoperative patients and degraded images. | Manually extracted from clinical report by one radiologist |
| Prevedello (2017) | CT, acute infarct  (‘algorithm 2’) |  | 71 CT head studies, from the training sample of algorithm 1, that did not have hemorrhage, mass effect or hydrocephalus, 46 with pathology and 25 healthy controls, with 2-D images split randomly into training (80%) and internal hold out test sets. Labels were extracted manually from clinical report. | 49 CT head studies from the same sample, that in practice would be the negative cases from algorithm 1. It was unexplained why only 19 normal cases were analysed by algorithm 2 when 47 of the normal cases were marked negative by algorithm 1. |  |
| Salehinejad (2021) | CT, ICH | CNN, ensemble model of modified ResNeXt-50 and ResNeXt-101 architectures, both pretrained on ImageNet.  Labels: slice-level Output: Binary prediction of ICH (present/not present) for each examination | 21,784 CT head studies, 1999-2018, 3 institutions (Brazil, USA), multiple scanner models. Slice level labels for 674,258 slices created by over 60 neuroradiologists for RSNA 2019 “Brain CT Hemorrhage Challenge”. | 5,965 consecutively acquired emergency CT head studies, Jan 2019 - Dec 2019, from 1 tertiary hospital, 3 models from same manufacturer. | Manually extracted from neuroradiologist report by research assistant - sample of 600 reviewed by radiologist with 100% examination-level labels correct, 98.1% correct for ICH subtype |
| Wang (2021) | CT, ICH | Ensemble model of CNN and two recurrent neural networks. Winner of the 2019-RSNA “Brain CT Hemorrhage Challenge”. Labels: slice-level Output: Binary prediction of ICH (present/not present) for each slice and examination | 19,530 CT head studies, 1999-2018, 3 institutions (Brazil, USA), multiple scanner models. Slice level labels for 674,258 slices created by over 60 neuroradiologists for RSNA 2019 “Brain CT Hemorrhage Challenge” | CQ500 (see Chilamkurthy, 2018) | CQ500 (see Chilamkurthy, 2018) |
| Voter (2021) | CT, ICH | Aidoc (see Buls, 2018) | Aidoc (see Buls, 2018) | 3,605 consecutively acquired CT head studies, July 2019 - Dec 2019, from 1 tertiary hospital, 7 scanners from same manufacturer | One neuroradiologist reviewed the clinical report and the AI prediction to set the ground truth. |
| Gauriau (2021) | MR, any pathology | CNN  Labels: Examination-level  Output: Binary prediction of pathology (present/not present) for each examination | 2,741 axial MR FLAIR examinations, consisting of 1,987 adults collected from 2007-2017 from 1 institution and 5,808 female patients only, including children, collected in 2017 from multiple sites at another institution. These datasets were originally collected for other studies. | 1,489 consecutive MR axial FLAIR examinations collected in 2019, children included, multiple sites but some data came from the same institution as training data, 4 scanner vendors, 14 scanner models | Manually extracted from reports, and images independently reviewed by a radiologist from another institution. Discrepancies would be settled by consensus. |
| Nael (2021) | MR, any pathology | CNN, modified U-net architecture  Input: MR FLAIR, ADC, DWI  Labels: Examination-level  Output: Binary prediction of pathology (present/not present) for each examination | 12,143 MR examinations of adult patients, after exclusion for 'non-definitive labels' (86) and without mandatory sequences (1,851), randomly split into training (9,845), internal validation (1,248) and internal hold-out test set (1,050). 2 scanner vendors, 19 scanner models | 1,072 MR examinations of adult patients, after exclusion for lack of mandatory sequences (659), 2 external institutions, 3 scanner vendors | One neuroradiologist extracted examination-level labels based on the clinical report |
| Nael (2021) | MR, ICH |  |  |  |  |
| Nael (2021) | MR, acute infarct |  |  |  |  |

CT = computed tomography, NCCT = non-contrast computed tomography, MR = magnetic resonance, FLAIR = fluid-attenuated inversion recovery, DWI = diffusion weighted imaging, ADC = apparent diffusion coefficient. FLAIR, DWI and ADC are commonly used MR sequences. ICH = intracranial hemorrhage, CNN = convolutional neural network, NLP = natural language processing, vRAD = Virtual Radiologic (commercial teleradiology solution), RSNA = Radiological Society of North America. Aidoc, Qure.ai and Avicenna.ai are commercial vendors of AI products. Aidoc v1.0, Aidoc v1.3 and CINA v1.0 are commercial AI solutions. CQ500 is a publicly available test dataset of CT head examinations from 491 patients. mask R-CNN, PatchFCN, GoogLeNet, ResNet18, ResNet38, ResNeXt-50, ResNeXt-101, U-net, DeepLab and DeepMedic are CNN architectures, published in academic literature. ImageNet is a large visual database often used in computer vision research; pretraining a model on ImageNet is a form of transfer learning.

# Supplementary Material 6: Further details for the assessment of risk of bias

## Patient Selection

Five studies (5/16, 31%) were considered overall to be at low risk of bias for patient selection ^12–16^.

Seven studies (7/16, 44%) that used temporal validation alone without external validation, were considered to have a high risk of bias for patient selection as there is limited assessment of generalisability ^17–23^, compared to 9/16 (56%) studies where AI models were externally validated on test data from other institutions ^12–16,24–27^.

Ten studies (10/16, 63%) with consecutive sampling of cases for test datasets ^12–18,20,21,23^ were considered to have a low risk of bias for this attribute. The sampling method was unclear in one study (1/16, 6%) ^27^. A high risk of bias was found for the remaining four studies (4/16, 25%) where target condition cases were added to enrich test datasets, which were therefore unrepresentative of the prevalence of abnormalities encountered in clinical practice ^19,24–26^. Similarly, there was a high risk of bias in one intracranial hemorrhage detection study (1/16, 6%) which excluded patients in the test dataset if the clinical request for the scan did not state the word “hemorrhage” ^22^; we considered this an inappropriate exclusion.

## Index test

Researchers can choose different “operating points”, by adjusting the threshold at which the continuous output of their AI model is converted into a binary decision for abnormality detection. This allows for the same model to be tuned to favour, for example, either sensitivity or specificity. Three studies (3/16, 19%) were considered at high risk of bias for selecting the operating point after testing as it allows researchers to present optimised accuracy metrics, and fails to convey the reliability of the AI model at the threshold chosen during model development, prior to testing ^19,24,25^. It was unclear whether an operating point has been selected before testing in 3/16 (19%) studies ^20,23,26^.

There were concerns regarding applicability in eight studies (8/16, 50%) as they assessed AI model performance in laboratory conditions (“analytical validation”^28^) only ^16,19,21,22,24–27^. In contrast, four studies (4/16, 25%) placed the AI model within the clinical pathway (“clinical validation”) ^12–14,17^, which more closely resembles a “real world” environment and therefore the intended applicability. In the remaining four studies (4/16, 25%), it was unclear whether the AI model was validated clinically or in laboratory conditions ^15,18,20,23^.

## Reference Standard

The reference standard in all studies was radiologist assessment. Studies that used fewer than two radiologists to assess the images of a scan for their reference standard were considered at high risk of bias, as individual radiologists do not have perfect accuracy. For example, one study reported that the agreement between two neuroradiologists to label MRI brain reports as normal or abnormal was 94.9% ^29^. Five studies (5/16, 31%) were therefore considered to have high risk of bias as only the clinical report was reviewed (in these cases, the report was assessed by either a single radiologist ^13,20,27^, research assistant ^15,17^ or an automated natural language processing algorithm ^17^). 10 (10/16, 56%) studies were at a low risk of bias for this attribute as at least two radiologists had reviewed the images. In 2/16 (13%) studies, a single radiologist assessed both the images and the clinical report written by a different radiologist ^12,23^. Of the remaining studies with a low risk of bias for this attribute, 3/16 (19%) used the “majority vote” of three independent radiologists as the reference standard ^24–26^ and 5/16 (31%) used a consensus of multiple radiologists (ranging from 2-4) ^14,18,19,21,22^. A high risk of bias was additionally found for one study (1/16, 6%) as the reference standard was informed by the output of the AI model (the index test) ^16^; the study was excluded from the meta-analysis due to this fundamental methodological flaw.

Of the three studies that aimed to detect any pathology, 2/16 (13%) did not consider healthy ageing leading to age-appropriate brain volume loss as normal ^30^ which raised concerns for applicability. We noted that there was a high prevalence of pathology in both test samples, 64% ^21^ and 81% ^27^ respectively, which may have been influenced by this decision. It was unclear how healthy aging was considered in one study (1/16, 6%); we note 72% of their test sample was considered abnormal ^18^.

## Flow and timing

There was a high risk of bias for flow and timing in two studies (2/16, 13%) where there was a discrepancy between the cases in the final analysis and the initial sample (e.g., due to AI model processing failures some patients were excluded from the final analysis) ^14,19^. There were discrepancies in two studies (2/16, 13%) between the published data and the published contingency tables without adequate explanation, which were also considered as having a high risk of bias ^17,20^. One study had two test datasets. One of the test datasets consisted of only 29% (140/491) of the CQ500 dataset without further explanation, therefore we excluded this dataset from analysis; we included the other test dataset which was from their own institution and was temporally distinct from their training data ^18^.

# Supplementary Material 7: Direct comparison to radiologists

The CQ500 dataset is a publicly available dataset of CT heads obtained from a representative clinical cohort and then enriched through the addition of intracranial hemorrhage cases, resulting in 205/491 cases of intracranial hemorrhage^24^. The reference standard for the CQ500 dataset is the “majority vote” from three radiologists independently reviewing each examination under laboratory conditions. When an AI model is undergoing validation and is being compared to the reference standard, there is also the opportunity for the AI model to be compared to the three individual radiologists, whose performance accuracy is also open source. Three studies (3/16, 19%) used the CQ500 dataset for AI model testing during external validation^24–26^. A statistical comparison was available for one study which showed equivalent sensitivity (p = 0.86) between radiologists and the AI model but with poorer specificity (p < 0.001)^24^.

In another study with a small test dataset of 200 cases containing 25 cases of intracranial hemorrhage, the performance of a temporally validated AI model^19^ was compared with four radiologists, who independently reviewed each examination under laboratory conditions. Separately, the consensus of two different neuroradiologists was used as the reference standard. No statistical comparison was available.

The performance of AI models and the individual radiologists from these four studies are highlighted in Figure 5.

England’s National Institute for Health and Care Excellence (NICE) aligns professional standards through clinical guidelines and offers evidence-based guidance globally^31^. The findings of our review, unlike those of NICE, did not show that AI products are as effective at detecting intracranial hemorrhages as neuroradiologists^32^. Direct comparison to radiologists was available in four intracranial hemorrhage detection studies. Only one externally validated AI model appeared to show comparable performance to individual radiologists, albeit under laboratory conditions^26^. Another study showed a statistically comparable sensitivity to pooled radiologists but with inferior specificity^24^. It should be noted however that evidence from mammography screening suggests that radiologist performance in the laboratory is significantly worse than in a clinical setting (the ‘laboratory effect’) which is thought to be due to the lower stakes involved^33^.

# Supplementary material 8: Commercial solutions

Many commercial AI solutions exist for brain imaging. In CT imaging, for example, 11 vendors provide commercially available (CE-marked) AI products for intracranial hemorrhage detection, but only four products have had their accuracy metrics published in peer-reviewed journals^34,35^. This lack of transparency is indicative of a wider problem in commercially available AI in medical imaging – a recent review found that of the available CE-marked AI products, only 36% had peer-reviewed evidence and 18% demonstrated clinical impact in the broadest sense^35^.

Of the commercially available solutions included in this review, Aidoc demonstrated consistent accuracy when clinically validated in three independent patient cohorts in Belgium and the US^12–14^. By comparison, studies validating AI from Qure.ai and Avicenna.ai provided their own external test datasets, which is a potential source of bias. Another potential source of bias is that the model trained by Qure.ai used a training dataset that was exclusively sampled within India – further validation in other countries would be required to demonstrate generalisability to other countries where it is marketed^24^. The model from Avicenna.ai was only trained on a subset of acute intracranial hemorrhage, and was only tested on a subset of CT head examinations that specifically stated the word “hemorrhage” in the clinical request^22^. Clinically, non-acute intracranial hemorrhage, often called “haematoma”, can share many of the same management implications as acute intracranial hemorrhage. Furthermore, confining the detection to a population with a high clinical suspicion of the target condition, also limits applicability in routine clinical practice as this is not representative.

# Supplementary Figures

Supplementary Figure 1: PRISMA flow diagram

Studies excluded from meta-analysis (n = 6):

Insufficient studies detecting same target condition within same modality (n = 5)

Severe methodological bias (n = 1)

Records excluded:

(n = 37,136)

Studies included for meta-analysis (n = 10)

Records identified from database searches:

MEDLINE Ovid (n = 19,370)

Embase Ovid (n = 28,451)

Cochrane library (n = 720)

Web of Science Core Collection (n = 21,478)

Additional records identified from other sources (n = 0)

Records removed *before screening*:

Duplicate records removed (n = 27,149)

Records screened against inclusion criteria (n = 42,870)

Full text assessed for eligibility

(n = 5,734)

Studies excluded (n = 5,718):

Publication type (e.g. conference proceedings)

(n = 1,434)

AI does not perform abnormality detection (n = 2,824)

Test dataset did not have combination of normal scans, scans with the target condition and scans with one or more non-target conditions (n = 1,239)

Interval validation results only without temporal or external validation (n = 218)

Accuracy metrics not provided at the patient level (n = 3)

Studies included for narrative synthesis

(n = 16)

AI models analysed in included studies

(n = 19)

**Identification of studies via all sources**

**Identification**

**Screening**

**Included**

Supplementary Figure 2: Diagnostic test accuracy of AI models in CT imaging in receiver operating characteristic (ROC) space, compared with individual radiologists. An ideal classifier would be at the top-left corner at (0, 1). The size of each marker is proportional to the size of the test dataset. In studies where there was more than one operating point, we chose the operating point with the highest sensitivity. ICH = intracranial hemorrhage. CQ500 = CQ500 external test dataset. Qure.ai, Aidoc and Avicenna.ai are commercial vendors for AI products.


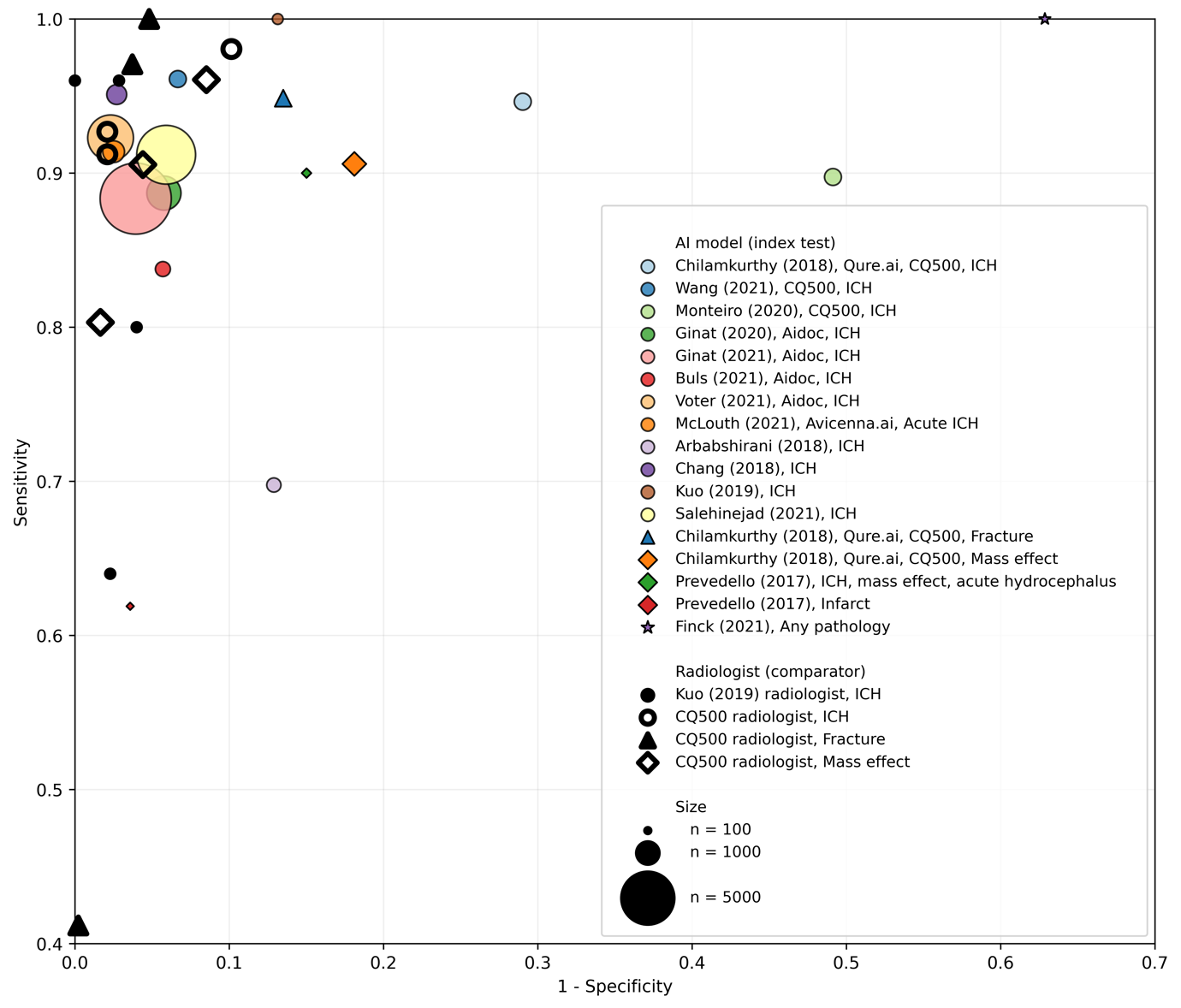


Supplementary Figure 3: Diagnostic test accuracy of AI models in MRI in ROC space. The size of each marker is proportional to the size of the test dataset. An ideal classifier would be at the top-left corner at (0, 1).


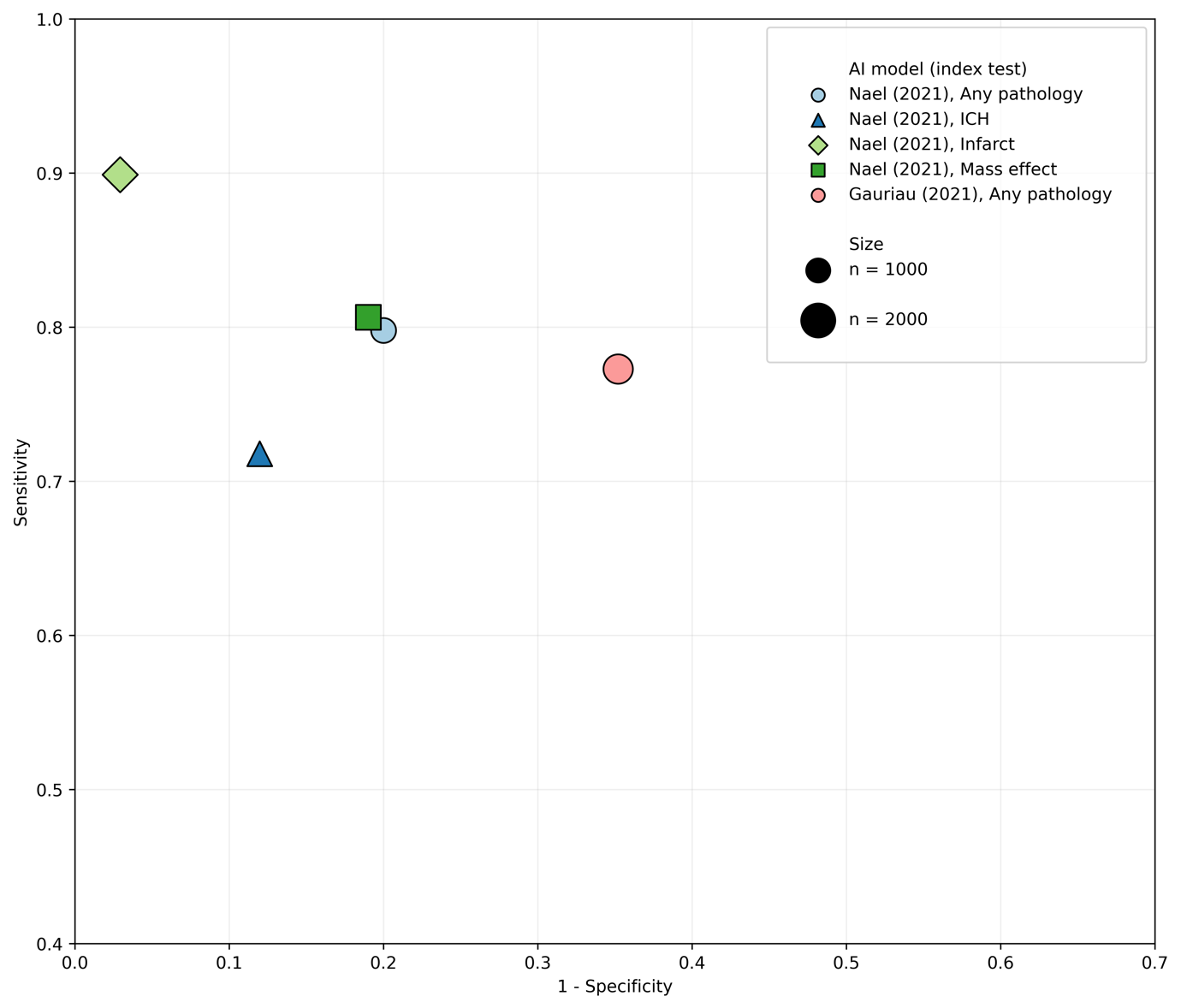


# References

1. Nussbaumer-Streit B, Klerings I, Dobrescu AI, et al. Excluding non-English publications from evidence-syntheses did not change conclusions: a meta-epidemiological study. *J Clin Epidemiol* 2020;118:42–54.

2. Whiting PF, Rutjes AWS, Westwood ME, et al. QUADAS-2: a revised tool for the quality assessment of diagnostic accuracy studies. *Ann Intern Med* 2011;155:529–36.

3. Mongan J, Moy L, Charles E. Kahn J. Checklist for Artificial Intelligence in Medical Imaging (CLAIM): A Guide for Authors and Reviewers. *Radiol Artif Intell* 2020;2:e200029.

4. Abu-Akel A, Bousman C, Skafidas E, et al. Mind the prevalence rate: overestimating the clinical utility of psychiatric diagnostic classifiers. *Psychol Med* 2018;48:1225–7.

5. Tenny S, Hoffman M. *Prevalence*. StatPearls Publishing; 2017.

6. Hocking KC, Wright CR, Alhun U, et al. Acute haemorrhage rate in 28,000 Out-of-Hours CT heads. *Br J Radiol* 2022;94:20210580.

7. Ebrahimzadeh S, Islam N, Dawit H, et al. Thoracic imaging tests for the diagnosis of COVID-19. *Cochrane Database Syst Rev* 2022;2022.

8. Reitsma JB, Glas AS, Rutjes AWS, et al. Bivariate analysis of sensitivity and specificity produces informative summary measures in diagnostic reviews. *J Clin Epidemiol* 2005;58:982–90.

9. Zwinderman AH, Bossuyt PM. We should not pool diagnostic likelihood ratios in systematic reviews. *Stat Med* 2008;27:687–97.

10. Doebler P. Mada: meta-analysis of diagnostic accuracy. 2015. [Epub ahead of print].

11. Tenny S, Hoffman M. *Prevalence*. StatPearls Publishing; 2017.

12. Ginat DT. Analysis of head CT scans flagged by deep learning software for acute intracranial hemorrhage. *Neuroradiology* 2020;62:335–40.

13. Ginat D. Implementation of Machine Learning Software on the Radiology Worklist Decreases Scan View Delay for the Detection of Intracranial Hemorrhage on CT. *Brain Sci* 2021;11:832.

14. Buls N, Watté N, Nieboer K, et al. Performance of an artificial intelligence tool with real-time clinical workflow integration--Detection of intracranial hemorrhage and pulmonary embolism. *Phys Medica Eur J Med Phys* 2021;83:154–60.

15. Salehinejad H, Kitamura J, Ditkofsky N, et al. A real-world demonstration of machine learning generalizability in the detection of intracranial hemorrhage on head computerized tomography. *Sci Rep* 2021;11:1–11.

16. Voter AF, Meram E, Garrett JW, et al. Diagnostic accuracy and failure mode analysis of a deep learning algorithm for the detection of intracranial hemorrhage. *J Am Coll Radiol* 2021;18:1143–52.

17. Arbabshirani MR, Fornwalt BK, Mongelluzzo GJ, et al. Advanced machine learning in action: identification of intracranial hemorrhage on computed tomography scans of the head with clinical workflow integration. *NPJ Digit Med* 2018;1:1–7.

18. Finck T, Schinz D, Grundl L, et al. Automated pathology detection and patient triage in routinely acquired head computed tomography scans. *Invest Radiol* 2021;56:571–8.

19. Kuo W, Hӓne C, Mukherjee P, et al. Expert-level detection of acute intracranial hemorrhage on head computed tomography using deep learning. *Proc Natl Acad Sci* 2019;116:22737–45.

20. Prevedello LM, Erdal BS, Ryu JL, et al. Automated critical test findings identification and online notification system using artificial intelligence in imaging. *Radiology* 2017;285:923–31.

21. Gauriau R, Bizzo BC, Kitamura FC, et al. A Deep Learning--based Model for Detecting Abnormalities on Brain MR Images for Triaging: Preliminary Results from a Multisite Experience. *Radiol Artif Intell* 2021;3:e200184.

22. McLouth J, Elstrott S, Chaibi Y, et al. Validation of a deep learning tool in the detection of intracranial hemorrhage and large vessel occlusion. *Front Neurol* 2021;12:655.

23. Chang PD, Kuoy E, Grinband J, et al. Hybrid 3D/2D Convolutional Neural Network for Hemorrhage Evaluation on Head CT. *Am J Neuroradiol* 2018;39:1609–16.

24. Chilamkurthy S, Ghosh R, Tanamala S, et al. Deep learning algorithms for detection of critical findings in head CT scans: a retrospective study. *Lancet* 2018;392:2388–96.

25. Monteiro M, Newcombe VFJ, Mathieu F, et al. Multiclass semantic segmentation and quantification of traumatic brain injury lesions on head CT using deep learning: an algorithm development and multicentre validation study. *Lancet Digit Heal* 2020;2:e314--e322.

26. Wang X, Shen T, Yang S, et al. A deep learning algorithm for automatic detection and classification of acute intracranial hemorrhages in head CT scans. *NeuroImage Clin* 2021;32:102785.

27. Nael K, Gibson E, Yang C, et al. Automated detection of critical findings in multi-parametric brain MRI using a system of 3D neural networks. *Sci Rep* 2021;11:1–10.

28. FDA-NIH Biomarker Working Group. BEST (Biomarkers, EndpointS, and other Tools) Resource. *BEST ( Biomarkers , EndpointS , other Tools ) Resour* 2016. [Epub ahead of print].

29. Wood DA, Kafiabadi S, Al Busaidi A, et al. Labelling Imaging Datasets on the Basis of Neuroradiology Reports: A Validation Study. *Lect Notes Comput Sci (including Subser Lect Notes Artif Intell Lect Notes Bioinformatics)* 2020;12446 LNCS:254–65.

30. Resnick SM, Pham DL, Kraut MA, et al. Longitudinal Magnetic Resonance Imaging Studies of Older Adults: A Shrinking Brain. *J Neurosci* 2003;23:3295–301.

31. NICE International.

32. National Institute for Health and Care Excellence (NICE). Artificial intelligence for analysing CT brain scans. *Medtech Innov Brief 207* 2020. [Epub ahead of print].

33. Gur D, Bandos AI, Cohen CS, et al. The “laboratory” effect: comparing radiologists’ performance and variability during prospective clinical and laboratory mammography interpretations. *Radiology* 2008;249:47–53.

34. AI for radiology: an implementation guide. 2022. [Epub ahead of print].

35. van Leeuwen KG, Schalekamp S, Rutten MJCM, et al. Artificial intelligence in radiology: 100 commercially available products and their scientific evidence. *Eur Radiol* 2021;31:3797–804.
